# Comparing metabolomic and pathologic biomarkers alone and in combination for discriminating Alzheimer’s disease from normal cognitive aging

**DOI:** 10.1186/2051-5960-1-28

**Published:** 2013-06-27

**Authors:** Alison A Motsinger-Reif, Hongjie Zhu, Mitchel A Kling, Wayne Matson, Swati Sharma, Oliver Fiehn, David M Reif, Dina H Appleby, P Murali Doraiswamy, John Q Trojanowski, Rima Kaddurah-Daouk, Steven E Arnold

**Affiliations:** 1 Bioinformatics Research Center, Department of Statistics, North Carolina State University, Raleigh, NC, USA; 2 Department of Psychiatry and Behavioral Sciences, Duke University, Durham, NC, USA; 3 Penn Memory Center, Department of Psychiatry, Perelman School of Medicine, University of Pennsylvania, Philadelphia, PA, USA; 4 Behavioral Health Service, Philadelphia VA Medical Center, Philadelphia, PA, USA; 5 Department of Systems Biochemistry, Bedford VA Medical Center, Bedford, MA, USA; 6 UC Davis Genome Center, University of California Davis, Davis, CA, USA; 7 Duke Institute for Brain Sciences, Duke University, Durham, NC, USA; 8 Center for Neurodegenerative Disease Research, Institute on Aging and Department of Pathology and Laboratory Medicine, Perelman School of Medicine, University of Pennsylvania, Philadelphia, PA, USA; 9 Department of Neurology, Perelman School of Medicine, University of Pennsylvania, Philadelphia, PA, USA; 10 Department of Psychiatry, Duke University Medical Center, Box 3950, Durham, NC 27710, USA

**Keywords:** Alzheimer’s disease, Dementias, Metabolomics, Cerebrospinal fluid biomarkers, Stepwise logistic regression

## Abstract

**Background:**

A critical and as-yet unmet need in Alzheimer disease (AD) research is the development of novel markers that can identify individuals at risk for cognitive decline due to AD. This would aid intervention trials designed to slow the progression of AD by increasing diagnostic certainty, and provide new pathophysiologic clues and potential drug targets.

**Results:**

We used two metabolomics platforms (gas chromatography-time of flight mass spectrometry [GC-TOF] and liquid chromatography LC-ECA array [LC-ECA]) to measure a number of metabolites in cerebrospinal fluid (CSF) from patients with AD dementia and from cognitively normal controls. We used stepwise logistic regression models with cross-validation to assess the ability of metabolite markers to discriminate between clinically diagnosed AD participants and cognitively normal controls and we compared these data with traditional CSF Luminex immunoassay amyloid-β and tau biomarkers. Aβ and tau biomarkers had high accuracy to discriminate cases and controls (testing area under the curve: 0.92). The accuracy of GC-TOF metabolites and LC-ECA metabolites by themselves to discriminate clinical AD participants from controls was high (testing area under the curve: 0.70 and 0.96, respectively).

**Conclusions:**

Our study identified several CSF small-molecule metabolites that discriminated especially well between clinically diagnosed AD and control groups. They appear to be suitable for further confirmatory and validation studies, and show the potential to provide predictive performance for AD.

## Background

Alzheimer’s disease (AD) is a major public health threat and there is an urgent need to validate biomarkers that are relevant to early detection and disease progression [1-9]. In the early clinical and "preclinical" stages of the illness, the symptoms of forgetfulness can be ambiguous and easily confused with the benign forms of memory loss that are associated with normal aging. A recent clinico-pathologic comparison study of more than 900 patients diagnosed at major centers in the United States found that 17-30% of clinical diagnoses were inconsistent with autopsy diagnoses [1]. There is a critical but unmet need for early detection and intervention of AD, as well as for identifying disease-modifying therapies that will slow the progression of dementia, delay the onset of dementia or prevent AD [2,4,6-9]. There is a need for objectively and precisely measurable biomarkers to complement clinical assessment methods such as cognitive and psychometric tests [3,5,8,9]. If they are developed, validated signatures for AD will enable a rapid identification of prodromal AD patients and could potentially enable the evaluation of drug responses, accelerating the whole process of drug development [2-4,6-8].

The development of AD biomarkers is complicated by the lack of diagnostic precision, difficulty in obtaining post-mortem verification, variability in clinical features and rates of progression, complex disease genetics and the multiple molecular pathways affected. Different types of biomarkers have been pursued for the detection of AD using imaging, genetic and biochemical tools [3,5,8-11]. Studies of blood markers have had difficulty capturing neurochemical changes consistently [9]. While dozens of cerebrospinal fluid (CSF) studies have been conducted in AD participants, such studies have usually either used a small sample size or examined only a few biochemical metabolites at a time due to logistic difficulties. The multicenter Alzheimer's Disease Neuroimaging Initiative study has enabled investigators to establish the utility of CSF Abeta and tau markers for discriminating AD from controls [5]. Neuroimaging methods (e.g., volumetric magnetic resonance imaging, amyloid positron emission tomography [PET] scans) offer promise as surrogate markers of neuronal loss and neuropathology, and the florbetapir F18 PET scan—for the detection of neuritic beta-amyloid plaques—just became available clinically as a diagnostic adjunct [8,10,11]. However, Abeta plaque is not specific for AD since it is also present in dementia with Lewy bodies, advanced Parkinson's disease and up to one-third of cases of normal aging [10,11]. There has been a lack of validated “mega” metabolic platforms that can simultaneously study the dozens of different biochemical pathways that may be affected in AD, both in relation to each other and in relation to clinical characteristics of the disease. Such platforms could potentially provide new insights, adding to those of single biomarker studies.

Metabolomics, the omics science of biochemistry, is a global approach to understanding the regulation of metabolic pathways and the metabolic networks of a biological system [12-23]. Metabolomics complements data derived from genomics, transcriptomics and proteomics to assist in providing a systems approach to the study of human health and disease. Metabolomics is the comprehensive study of the metabolome, the repertoire of bio-chemicals (or small molecules) present in cells, tissue and/or body fluids. The metabolome defines a metabolic state as regulated by net interactions between genetic and environmental influences, and provides information that can possibly bridge the gap between genotype and phenotype. Metabolomics provides powerful tools to map perturbations in biochemical pathways and networks in disease and in response to treatment [15,16]. For example, we have identified major changes in the norepinephrine pathway in patients with AD [24] and also changes in the ceramide-sphingomyelin pathway [25]. We have mapped several pathways implicated in schizophrenia and in response to antipsychotics [26-30]. In depression, we have identified changes in neurotransmitter pathways, lipid metabolism and beta-oxidation among other changes [31-33], and we were able to demonstrate that a patient’s metabolic profile (metabotype) enables the prediction of response to the antidepressant sertraline [34].

Recently, we applied metabolomics approaches to profile CSF derived from a cohort of clinically diagnosed AD patients and cognitively normal controls to map global biochemical changes in AD. We have employed a liquid chromatography LC-ECA array (LC-ECA) metabolomics platform to identify metabolites within key neurotransmitter pathways of tryptophan, tyrosine and purine that are implicated in the pathogenesis of AD [35]. This platform has been extremely useful for the study of central nervous system diseases and their treatment [25,29,30,36-38]. Since different profiling methodologies have distinct advantages for conducting metabolomic studies and cover different areas of biochemistry, in this paper we incorporate an additional platform for our analyses: the gas chromatography-time of flight (GC-TOF) mass spectrometry. GC-TOF enables the interrogation of over 160 metabolites of intermediary metabolism and provides an overview of metabolic changes. We build classification models for the disease phenotype with different combination of metabolomics and pathogenesis variables to derive initial estimates of diagnostic utility of different types and combinations of predictors.

## Methods

### Participants

Metabolomic, protein and genetic data for the current study were gathered from a cross-section of participants who were recruited and evaluated in clinical research by the Penn Memory Center [10]. Most of these participants were enrolled in a prospective multi-site longitudinal biomarker study and were also included in our prior report focusing on LC-ECA metabolites [35]. Forty AD patients and 38 controls with banked CSF samples were analyzed for this report. Written informed consent was collected as appropriate, and all protocols were approved by the University of Pennsylvania and the Duke University Medical Center institutional review boards.

### Inclusion and exclusion criteria

For the AD subgroup, subjects had to meet National Institute of Neurological, Communicative Disorders and Stroke–Alzheimer Disease and Related Disorders Association criteria for probable or possible AD; all but one patient were classified as having mild to moderate dementia based on combination of clinical judgment, Mini Mental State Exam (MMSE) [39] score and Functional Rating Scale (FRS) [40] score. Participants could be on stable approved therapies. Participants were excluded from this group if they had a history of clinically meaningful stroke, Parkinson's disease, untreated current major depression, psychosis or a primary diagnosis of a non-AD dementia.

To be included in the cognitively normal subgroup, the following inclusion criteria had to be met: 1) No significant cognitive impairment verified by psychometric testing norms, and 2) No significant change in functional abilities verified by a knowledgeable informant. Participants were excluded if they had a history of significant stroke, current untreated major depression, psychosis, mild cognitive impairment (MCI) or dementia. Subjects in both groups had to be over 65, have a reliable informant and consent to longitudinal follow up.

Diagnostic assessments were generally made in a consensus conference after comprehensive neurologic, physical and neuropsychological testing was performed. Most patients had multiple psychometric tests, including the Clinical Dementia Rating, the Dementia Rating Scale-Second Edition (DRS-2), the MMSE, and tests of frontal executive function, memory, language, praxis, visuospatial construction, motor performance, mood and function. MMSE scores were not always available at the time of baseline blood collection but the nearest available MMSE was used for staging purposes along with function and clinical judgment.

### CSF collection

Baseline CSF samples obtained in polypropylene tubes were utilized for metabolomics. CSF was obtained, as described previously [5], by lumbar puncture using an atraumatic Sprotte needle in most cases. To minimize contamination from blood associated with needle insertion, the first 1-2 ml of CSF (or more if needed) were discarded and the next 20 ml were aliquoted into 0.5 ml portions, bar coded and stored in a -80°C freezer until processing. The standardized Luminex multiplex assay technique for amyloid beta 1-42 (Ab42), total tau (t-tau) and tau phosphorylated at the threonine 181 position (p-tau) are described elsewhere [5]. Aliquots were shipped overnight on dry ice for metabolomics processing.

### Metabolomics profiling: LC-ECA

The LC-ECA platform that has been extensively used and validated in our prior studies in neurodegenerative and psychiatric disorders [17,34,41-43]. The method is specific for compounds that will undergo LC-ECA oxidation or reduction, and includes multiple compounds from the tyrosine, tryptophan, sulfur amino acid and purine pathways, as well as markers of oxidative stress and protection (see Additional file 1: Table S1). At the time of preparation, a pool was created from equal amounts of small aliquots of each study sample, which was treated identically to a sample. The pooled samples were run after every six study samples, followed by a known standards mix to ensure uniformity along the length of the run. Metabolite peak identification was carried out using the CEAS software (ESA, Inc., Chelmsford, MA). The main metabolite peaks of knowns and unknowns were aligned and relative concentrations to a central CSF sample pool (taken at 100%) were measured. These peak-tables were used for the subsequent statistical analysis where we focused on 71 total metabolites, of which 24 were known compounds (Additional file 1: Table S1).

### GC-TOF mass spectrometry

CSF samples were aliquoted and maintained at -80°C until use, at which point 30 μl of CSF samples were thawed, extracted and derivatized. Briefly, 15 μl aliquots were extracted with 1 ml of degassed acetonitrile:isopropanol:water (3:3:2) at -20°C, centrifuged and decanted with subsequent evaporation of the solvent to complete dryness. A clean-up step with acetonitrile/water (1:1) removed membrane lipids and triglycerides, and the supernatant was again dried down. Internal standards C8-C30 fatty acid methyl esters were added and the sample was derivatized with methoxyamine hydrochloride in pyridine and subsequently by N-Methyl-N-(trimethylsilyl)trifluoroacetamide (MSTFA) (Sigma-Aldrich) for trimethylsilylation of acidic protons.

A Gerstel MPS2 automatic liner exchange system was used to inject 1 μl of sample at 50°C (ramped to 250°C) in splitless mode with a 25-second splitless time. An Agilent 6890 gas chromatograph (Santa Clara, CA) was used with a 30 m long, 0.25 mm i.d. Rtx5Sil-MS column with 0.25 μm 5% diphenyl film; an additional 10 m integrated guard column was used (Restek, Bellefonte PA). Chromatography was performed at a constant flow of 1 ml/minute, ramping the oven temperature from 50°C for to 330°C over 22 minutes. Mass spectrometry used a Leco Pegasus IV time of flight mass spectrometer with a 280°C transfer line temperature, electron ionization at -70 V and an ion source temperature of 250°C. Mass spectra were acquired from m/z 85-500 at 20 spectra/second and 1750 V detector voltage. Result files were exported to our servers and further processed by our metabolomics BinBase database. All database entries in BinBase were matched against the Fiaehn mass spectral library of 1,200 authentic metabolite spectra using retention index and mass spectrum information or the NIST05 commercial library. Identified metabolites were reported if present with at least 50% of the samples per study design group (as defined in the SetupX database [44]). Quantitative data were normalized to the sum intensities of all known metabolites and used for statistical investigation. Data on a total of 299 metabolites were collected using the MS platform.

### Statistical methods

Statistical analysis was performed in two stages to find variables that discriminate between AD participants and controls. First, univariate analyses were performed to understand the potential associations between the covariates collected and the disease. Importantly, we investigated the use of AD treatment drugs (cholinesterase inhibitors and memantine), as well as antidepressants, antipsychotics, anxiolytics, corticosteroids and statins to identify metabolites that might potentially be associated with drug metabolism and/or response. Second, multivariate modeling was performed using different combinations of data types (metabolomic, proteins, etc.) to evaluate the potential discriminatory power of each of these data types alone and in combination for predicting AD. To evaluate the predictive potential of these variables, the models were evaluated using cross-validation to assess the predictive performance of the models and further refine the variable included in a prediction analysis, so that the resulting models were evaluated on the testing data, as opposed to the training data.

#### Univariate analysis

Fisher’s exact tests were performed to examine the potential association of gender, race, and the use of different classes of drugs with disease status; two-sample t tests were used to test the difference in education, age and MMSE score between the two diagnostic groups.

#### Model building to evaluate the discrimination potential of variables

To evaluate the discriminatory power of the metabolites and compare to the Luminex values, we built predictive models of AD vs. control status. Prior to model-building analysis, the raw metabolite values were visually inspected by quantile-quantile normal plots to assess normality. Metabolites were log-transformed to improve normality. Metabolites were also filtered to prevent any potential confounding with the drug therapies used to treat the disease. This was done because different drugs are used in AD participants than in controls, and it is expected that metabolite profiles could change in response to treatment. Then, for the nominally significantly associated drugs, all metabolites were tested for association with drug status/use using Kruskal-Wallis tests. Those metabolites that were even nominally associated with drug status (p < 0.05) were filtered out prior to model building as they may potentially be related to drug metabolism and/or response. While this may be overly conservative, we wanted to be certain that any potential discrimination gained by adding the metabolites into a model was not confounded by drug response/metabolism. Additionally, metabolites were tested for association with both the ApoE genotype, with genotype coded as high risk and low risk groups (where E3/E4 an E4/E4 genotypes were high risk and all others were considered low risk) using Kruskal-Wallis tests of association. Again, nominally associated metabolites were removed prior to model building to prevent confounding with risk genotype.

Once the metabolites were filtered for independence from drug use and genotype, forward step-wise logistic regression was performed using a Bayesian Information Criterion (BIC) for variable selection and modeling with several different sets of variables. First, models were built using each of the following types of variables alone to evaluate the maximal potential prediction from each set of variables: Luminex proteins, LC-ECA metabolites, and GC-TOF metabolites. Second, model building was performed using all possible two-way combinations of variables (e.g., Luminex proteins plus the LC-ECA metabolites, Luminex proteins plus the GC-TOF metabolites, etc.). Next, models were built with all possible three-way combinations of variables. Finally, modeling was performed with all possible predictive variables.

In order to assess the predictive performance of the metabolites (and to limit potential overfitting), the model building was performed using five-fold cross-validation to evaluate the stability of the variable selection and model fit. The step-wise modeling approach was repeated for every 4/5 split of the data, and the variables included in the model were recorded along with a training AUC and a testing AUC (calculated on the 1/5 of the data left out of model building). In each cross-validation interval, the variables included were recorded, as the final model was selected based on cross-validation consistency (picking the variable[s] that were selected in the most cross-validation models). By using cross-validation, we were able to estimate a prediction error from the withheld data used in the validation process. While such an analysis is not as powerful for assessing the true predictive performance of a model, it is well established that k-fold cross-validation provides an estimate of the predictive performance, and k = 5 is considered a reasonable compromise between bias and variance for this estimate [45-47].

The predictive performance of the resulting models was evaluated using area-under the curve (AUC) values. Because of the high dimensional and sparse nature of the data, to try to assess whether the resulting models were better than would be expected by chance, we performed permutation testing to ascribe statistical significance to the resulting models. One thousand permuted datasets were generated, randomizing the case status at the same proportions as in the real data, and the entire data analysis procedure was repeated. The best correlation coefficient and AUC for each permuted dataset was recorded, and an empirical distribution of model fit statistics was generated across the 1000 permuted datasets. Then the values from the real data analysis were compared to the empirical distribution to generate an empirical p-value.

To test whether there were significant differences in the predictive performances of the resulting models (i.e., whether the differences were just by chance or were likely to represent true differences), DeLong’s tests were performed between the different models. A Bonferroni correction for the number of tests performed was used to determine the alpha level for significance for these AUC comparisons.

Finally, we used Pearson correlation analyses to test for correlation of the best metabolites (from the final predictive model) and MMSE scores.

## Results

### Demographic differences

Tests that compared clinical and demographic variables showed the AD and control participants to be generally well matched for age and gender. Among these variables, the only significant differences between groups were a higher educational level for controls (though this association was only nominally significant) and the use of two disease treatment drugs, cholinesterase inhibitor and/or memantine, in a subset of AD participants (Table 1).

**Table 1:** Participant demographics and clinical characteristics

**Characteristics**	**AD**	**CN**	**p-value**	**Test**
	**(N = 40)**	**(N = 38)**		
Mean Age +/- SD	69.0 +/- 9.1	69.5 +/- 9.7	0.825	T
Mean Years of Education +/- SD	14.8 +/- 3.6	16.6 +/- 3.0	0.015	T
Mean MMSE +/- SD	19.9 +/- 7.7	29.2 +/- 1.3	1.0E-9	T
% Male	25.0	34.21	0.459	F
% Caucasian	82.5	86.8	1.00	F
% Taking Antidepressant	30.0	21.05	0.441	F
% Taking Antipsychotic	5.0	0.0	0.494	F
% Taking Anxioytic	12.5	13.2	1.00	F
% Taking Corticosteroids	2.5	13.2	0.104	F
% Taking Cholinesterase Inhibitor	37.5	0.0	<1.0E-9	F
% Taking Memantine	15.0	0.0	0.026	F
% Taking Statins	20.0	26.3	0.595	F

Abbreviations: *AD* Alzheimer’s disease, *CN* Normal cognition, *SD* Standard Deviation, *T* two-sample *t* test, two-sided, *MMSE* Mini-Mental State Exam obtained at date closest to sample if not available concurrently, *F* Fisher’s exact test, two-sided.

### Drug associations

The results of the tests of association for the metabolites against drug use resulted in 134 that were nominally associated. Additionally, two metabolites were nominally associated with ApoE genotype status. Metabolites’ associations and their p-values from the drug association analysis are listed in Additional file 2. A list of the association results for each metabolite and ApoE genotype status are listed in Additional file 3. These metabolites were removed from the list of potential predictors for the next stage of analysis, leaving a total of 238 metabolites evaluated in the model building step.

### Model building

The summary of the model fits from the stepwise logistic regression modeling for the AD vs. control is listed in Table 2. The final models are listed in Additional file 4, with the logistic regression equation (with parameter estimates and included variables listed) for each cross-validation interval. All models were statistically significant according to the results of the permutation testing (p < 0.05 in all cases). The results of the Delong’s test comparisons of the discrimination of the models are shown in Table 3, including the results for all two-way combinations of resulting models. Only the statistically significant p-values (using a Bonferroni correction) are listed.

**Table 2:** Summary measures of model fit for each resulting model for discriminating between AD vs. controls in the full dataset

**Model variables**			**Model variable abbreviations**	**AUC**	**Sensitivity**	**Specificity**
**E**	**M**	**P**				
X			E	0.96	1.00*	0.90
	X		M	0.70	0.68	0.67
		X	P	0.92	0.97	0.83
X		X	P|E	0.90	0.93	0.89
	X	X	P|M	0.89	0.88	0.88
X	X	X	P|M|E	0.90*	0.92	0.89

These results are the average measures of model fit in the testing (not training) sets across all cross validation intervals. The variables included in the modeling process are highlighted in indicator columns, along with their abbreviations. The data types are Luminex amyloid and tau-related proteins (P), GC-TOF mass spectrometry metabolites (M) and LC-ECA metabolites (E).

Abbreviations: *AD* Alzheimer’s disease, *AUC* Area under the curve. *rounded.

**Table 3:** Results from the comparisons of prediction models built on Luminex phosphorylated proteins (P), GC-TOF mass spectrometry metabolites (M) and LC-ECA metabolites (E)

**Data types**	**AUC Test**					
	**E**	**M**	**P**	**P|E**	**P|M**	**P|M|E**
E		----	----	----	----	----
M	0.005		----	----	----	----
P		0.004		----	----	----
P|E		0.005			----	----
P|M	.045	0.005				----
P|M|E		0.004				

Abbreviations: *AD* Alzheimer’s disease, *AUC* Area under the curve.

As expected, the model built with CSF Aβ, t-tau and p-tau levels as measured in Luminex immunoasssays showed good discrimination of AD versus controls with an average testing AUC of 0.92 (Table 2). The model with the LC-ECA metabolites was also highly discriminatory with an average testing AUC of 0.96, slightly higher than the model built with the Luminex proteins. Remarkably, this discrimination was achieved with two metabolites that consistently were included in each cross-validation interval (5/5 cross-validation consistency): 15-65.533 and 8-93.65 the identities of which are currently unknown. By comparison, the GC-TOF mass spectrometry metabolites resulted in a model with much lower predictive performance (average testing AUC of 0.70) than the LC-ECA metabolites or Luminex proteins. Combining metabolomics with pathology markers did not increase accuracy much more and in some combinations reduced accuracy. To visually discriminate the predictive power of the models, the average performance (sensitivity and specificity) for each resulting model is depicted in Additional file 5: Figure S1. In this figure, better performance is seen as a lift in the models to the upper left quadrant of the graph. The models have been colored according to whether they used the metabolite variables in the model (red) or did not (blue).

**Figure 1:**
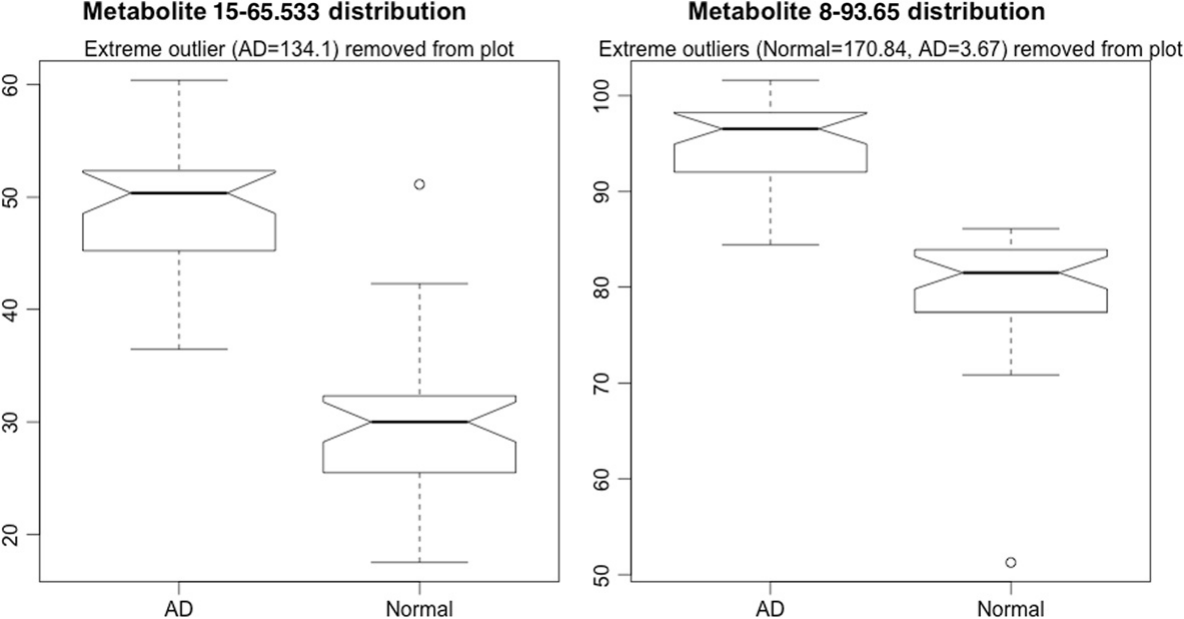
**Box-plot distributions of metabolites 15-65.533 and 8-93.65 for Alzheimer’s disease (AD) participants and controls, in relative concentrations.** The boxplots show the median and quartiles of each distribution, with outliers marked with dots. Outliers were defined as values more than 1.5 times the interquartile range from the median.

Since metabolites 15-65.533 and 8-93.65 had the most consistent association, we show in Figure 1 the distribution values of these metabolites for AD and control participants. It can be clearly seen that both of them are elevated in AD. Our correlation analysis indicated that there was only weak correlation between these metabolites and the MMSE score (not statistically significant).

## Discussion

These results show that in analyzing CSF, mapping the concentrations of small molecules in the LC-ECA platform produces initial prediction levels similar to those produced by well validated CSF neuropathology-related protein biomarkers for discriminating between clinically diagnosed AD and cognitively normal participants. Further, our data suggests that metabolomics markers alone may offer relatively high discriminatory ability to distinguish the clinical AD phenotype from controls, and the lack of correlation with MMSE suggests the markers may have potential applicability also in mild stages where there is relatively high need for accurate markers.

The current sample size is limited in its ability to truly build and evaluate potential predictive models without further evaluation and validation in larger studies; however, it is interesting that a model from a predictive resampling scheme with only 2 metabolites could be built that has high predictive potential. These results were found with a conservative screening approach that removed metabolites that are potential confounders due to differential drug response and also ApoE status.

While the potential predictive performance of the 15-65.533 and 8-93.65 metabolites is promising, their unknown identity makes biological interpretation difficult. This limits current interpretability, but it does demonstrate the potential of new, high-throughput technologies to identify novel metabolite markers that may be of biological and clinical relevance. Studies are in progress to further characterize and identify them, from both a bioinformatics perspective and a biochemical perspective. This will be helpful in enhancing our understanding of pathophysiologic mechanisms and the interrelationships between small molecules and AD-associated proteins.

Initial analyses to explore correlations with 15-65.533 and 8-93.65 show that they are significantly associated with several known metabolites that may help reveal their biological relevance (Additional file 6: Table S1). The correlation structure and pathway analyses of the electrochemistry metabolites collected in the current data are thoroughly discussed in a previous manuscript [35]. Correlations of the two unknowns with other metabolites in the study were tested in a post-hoc analysis to try to interpret the potential biological relevance of the unknown metabolite (with all metabolites, including those that were associated with drug response). For example, 15-65.533 is highly correlated with methionine (P = 1E-6), which is also correlated with disease status. Additionally, 15-65.533 is also correlated with several other known metabolites, including Indole-3-propionic acid (I-3-PA), Kynurenine (KYN), Indole-3-Acetic Acid and Guanosine. While the mechanisms and potential pathway relationships need to be evaluated in future studies, there is previous evidence of the involvement of these known metabolites in disease etiology. For example, I-3-PA has been shown to be involved in neuronal damage and oxidative stress in the brain [48,49], KYN is a major route of tryptophan metabolism and has been implicated in pathogenesis of several neuropsychiatric diseases. Several metabolites within this pathway were involved in the pathogenesis of AD in previous studies [50,51]. The other unknown metabolite, 8-93.65, is strongly correlated with MET (P = 3E-12) and glutathione (GSH, P = 3E-5), both of which are elevated in AD [35]. MET is the precursor for homocysteine and cysteine, which plays a critical role in GSH synthesis. The potential link of this pathway with AD along with details of the data analysis has been discussed in our previous manuscript [35].

In addition to the disease-metabolite associations, we also identified drug-associated metabolites. While not the primary goal of the current study, these associations may reveal interesting biology of drug metabolism and/or response. Again, as with the disease-associated metabolites, the majority of the drug-associated metabolites are compounds of unknown structure. Interestingly, one of the known metabolites, I-3-PA, which was associated with drug use, was associated with 15-65.533, potentially indicating/reinforcing a shared mechanism between the drug targets and disease etiology. This supports the potential of the metabolites to identify potential new drug targets by revealing insights into disease susceptibility.

We also identified two metabolites that were associated with ApoE genotype;

Phosphoethanolamine is an ethanolamine derivative that is used for synthesis of sphingomyelins that we previously implicated in AD [25], and monopalmitin (glycerol 1-palmitate) a lipid implicated in membrane integrity and stability and an energy storage source.

Should our findings be replicated and validated in a larger study of pathologically confirmed AD, it may lead to a clinically useful test. Similar studies in patients with MCI are underway to determine whether these combined models are capable of identifying distinct subgroups of MCI patients. Longitudinal follow-up of MCI patients can then determine whether the “AD-like” biomarker profile predicts the progression of cognitive decline and thus identifies a subgroup at high risk for developing dementia. Such participant groups are likely candidates for clinical trials of agents for slowing the progression of cognitive decline. In addition, these studies will also need to be extended to patients with other types of dementias, such as frontal temporal lobar dementia, Parkinson’s disease dementia and Lewy body dementias, to assess the specificity of these metabolites to pathologic AD vs. other dementias.

In addition to the above-stated limitations of sample size and current interpretability of results with metabolites of unknown structure, our AD participants were clinically diagnosed and we therefore did not have autopsy confirmation as a gold standard. Since our objective was to compare the performance of metabolomics markers against standard CSF Ab42 and tau measures, we did not use these measures to define pathologic AD. Therefore, it is likely that about 20% of our AD participants did not meet criteria for pathologic AD and about 20% of controls might harbor preclinical disease, which perhaps explains the observed accuracy of standard Ab42 and tau markers.

Additionally, the lack of correlation between the metabolites selected in the prediction models and MMSE highlights the potential of these metabolites as predictors of overall clinical AD diagnosis, and not necessarily a specific stage. Given our sample included both mild and moderate severity patients, studies of just mildly impaired patients would be more informative of the utility of the markers for early AD. Future studies should investigate these complex relationships, and interrogate potential associations amongst metabolites and other clinical and biological mediators and contributors to mild stages of AD.

## Conclusion

In conclusion, our preliminary results suggest that in analyzing CSF, some metabolomics variables have a high discriminatory ability to distinguish patients with clinical AD from cognitively normal controls. Further, our results show the potential of metabolite variables to provide discrimination potential of the same magnitude as the CSF amyloid and tau proteins.

## Competing interests

Drs. Kaddurah-Daouk, Doraiswamy, Zhu and Matson are inventors on patents in the metabolomics field. Dr. Kaddurah-Daouk has received funding from pharmaceutical companies for metabolomic studies. Dr. Arnold has received research grant support through the University of Pennsylvania from the NIH, the American Health Assistance Foundation, the Marian S. Ware Alzheimer’s Program, and several pharmaceutical companies and advisory/speaking honoraria from Universities, pharmaceutical companies and law firms. Dr. Trojanowski may accrue revenue in the future on patents submitted by the University of Pennsylvania wherein he is co-Inventor and he received revenue from the sale of Avid to Eli Lily as co-inventor on imaging related patents submitted by the University of Pennsylvania while he receives research support from the NIH, Bristol Myer Squib, AstraZeneca and several non-profits. Dr. Doraiswamy has received research grants (through Duke) and advisory/speaking fees from the NIH, the Alzheimer’s Association, the Alzheimer’s Drug Discovery Foundation, the Alzheimer’s Foundation of America, and several pharmaceutical companies; and owns stock in Sonexa and Clarimedix. Dr. Motsinger-Reif has received consulting honoraria from pharmaceutical companies. The remaining co-authors have no conflicts of interest.

## Authors’ contributions

RKD and SEA are PIs of study, conceived and designed the experiments, and contributed to interpretation of findings and writing of manuscript. AAM analyzed the metabolomics data and drafted the manuscript. HZ analyzed the data and contributed to the interpretation of results and writing of the manuscript. MAK, PMD and JQT contributed to the design of experiment and the interpretation of results. WM, SS, and, OF contributed metabolic analysis tools. DMF analyzed data. DHA contributed to data pre-processing and analysis. All authors read and approved the final manuscript.

## Authors’ information

Alison A Motsinger-Reif and Hongjie Zhu: Co-first authors.

Rima Kaddurah-Daouk and Steven E Arnold: Co-last authors.

## Supplementary Material

Additional file 1: Table S1 List of known compounds quantified by the LCECA platform. Click here for file

Additional file 2 Metabolites’ associations and their p-values from the drug association analysis. Click here for file

Additional file 3 Metabolites’ association with ApoE genotype status. Click here for file

Additional file 4 Final logistic regression models for the AD vs. control. Click here for file

Additional file 5: Figure S1 Average sensitivity (vertical axis) and 1-specificity (horizontal axis) for the stepwise logistic regression models across all cross-validation intervals for the Alzheimer’s disease vs. control and normal modeling, considering all combinations of data types. The data types are the phosphorylated proteins (P), GC-TOF mass spectrometry metabolites (M) and LC-ECA metabolites (E). Measured metabolite variables (M, E) and combinations that include these variables are shown in red. Click here for file

Additional file 6: Table S2 Known metabolites that are associated with 8-93.65 and/or 15-65.533. See Additional file 1 for abbreviations. Click here for file
