# Supplementary material for: Comparing metabolomic and pathologic biomarkers alone and in combination for discriminating Alzheimer’s disease from normal cognitive aging
Source: Acta Neuropathol Commun. 2013 Jun 27;1:28. doi: 10.1186/2051-5960-1-28 (PMC3893491; doi:10.1186/2051-5960-1-28)
Supplement: Additional file 1: Table S1 — List of known compounds quantified by the LCECA platform. [file 2051-5960-1-28-S1.docx]

**Table S1: List of known compounds quantified by the LCECA platform.**

| **Metabolite by Pathways** | **Abbreviation** | **Metabolite by Pathways** | **Abbreviation** |
| --- | --- | --- | --- |
| **Tryptophan** | | **Purine** | |
| Tryptophan | TRP | Guanosine | GR |
| 5-Hydroxyindoleacetic acid | 5-HIAA | Hypoxanthine | HX |
| 5-Hydroxytryptophan | 5-HTP | Uric acid | URIC |
| Kynurenine | KYN | Xanthine | XAN |
| Indole-3-acetic acid | I-3-AA | Xanthosine | XANTH |
| **Tyrosine** |  | Paraxanthine | PXAN |
| 4-Hydroxyphenylacetic acid | 4-HPAC | **Cysteine and Methionine** |  |
| Homovanillic acid | HVA | Glutathione (reduced) | GSH |
| Methoxyhydroxyphenlyglycol | MHPG | Methionine | MET |
| Tyrosine | TYR | **Other** |  |
| Vanillylmandelic acid | VMA | Ascorbic acid | ASA |
| **Phenylalanine** |  | Delta-tocopherol | DTOCO |
| 4-Hydroxybenzoic acid | 4-HBAC | Indole-3-propionic acid | I-3-PA |
| 4-Hydroxyphenyllactic acid | 4-HPLA |  |  |
| 2-Hydroxyphenylacetic acid | 2-HPAC |  |  |
