# Supplementary figures and images for: Comparing metabolomic and pathologic biomarkers alone and in combination for discriminating Alzheimer’s disease from normal cognitive aging
Source: Acta Neuropathol Commun. 2013 Jun 27;1:28. doi: 10.1186/2051-5960-1-28 (PMC3893491; doi:10.1186/2051-5960-1-28)

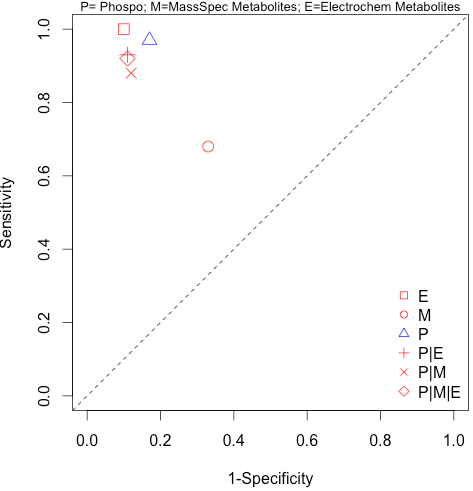

Supplement: Additional file 5: Figure S1 — Average sensitivity (vertical axis) and 1-specificity (horizontal axis) for the stepwise logistic regression models across all cross-validation intervals for the Alzheimer’s disease vs. control and normal modeling, considering all combinations of data types. The data types are the phosphorylated proteins (P), GC-TOF mass spectrometry metabolites (M) and LC-ECA metabolites (E). Measured metabolite variables (M, E) and combinations that include these variables are shown in red. [file 2051-5960-1-28-S5.tiff]
