# Supplementary material for: Comparing metabolomic and pathologic biomarkers alone and in combination for discriminating Alzheimer’s disease from normal cognitive aging
Source: Acta Neuropathol Commun. 2013 Jun 27;1:28. doi: 10.1186/2051-5960-1-28 (PMC3893491; doi:10.1186/2051-5960-1-28)
Supplement: Additional file 6: Table S2 — Known metabolites that are associated with 8-93.65 and/or 15-65.533. See Additional file 1 for abbreviations. [file 2051-5960-1-28-S6.docx]

**Table S2: Known metabolites that are associated with 8-93.65 and/or 15-65.533. See Additional File 1 for abbreviations.**

|  | Known  Metabolites | Correlation coefficient | P-value | Q-value |
| --- | --- | --- | --- | --- |
| 8-93.65 | MET | 0.6 | 2.5E-12 | 1.1E-10 |
|  | GSH | 0.38 | 0.000029 | 0.0002 |
|  | 5-HIAA | 0.34 | 0.00025 | 0.0012 |
|  | TYR | 0.32 | 0.00058 | 0.0024 |
|  | TRP | 0.31 | 0.00089 | 0.0033 |
|  | 4-HBAC | 0.29 | 0.0018 | 0.0057 |
|  | VMA | 0.21 | 0.027 | 0.044 |
| 15-65.533 | I-3-PA | 0.54 | 5.4E-10 | 1.6E-08 |
|  | MET | 0.44 | 0.000001 | 0.000012 |
|  | KYN | 0.36 | 0.000067 | 0.00037 |
|  | I-3-AA | 0.26 | 0.0047 | 0.013 |
|  | GR | 0.24 | 0.0093 | 0.021 |
